# Application of a Novel Grey Self-Memory Coupling Model to Forecast the Incidence Rates of Two Notifiable Diseases in China: Dysentery and Gonorrhea

**DOI:** 10.1371/journal.pone.0115664

**Published:** 2014-12-29

**Authors:** Xiaojun Guo, Sifeng Liu, Lifeng Wu, Lingling Tang

**Affiliations:** 1 School of Science, Nantong University, Nantong, Jiangsu Province, China; 2 College of Economics and Management, Nanjing University of Aeronautics and Astronautics, Nanjing, Jiangsu Province, China; 3 School of Electrical and Computer Engineering, Cornell University, Ithaca, New York, United States of America; The University of Tokyo, Japan

## Abstract

**Objective:**

In this study, a novel grey self-memory coupling model was developed to forecast the incidence rates of two notifiable infectious diseases (dysentery and gonorrhea); the effectiveness and applicability of this model was assessed based on its ability to predict the epidemiological trend of infectious diseases in China.

**Methods:**

The linear model, the conventional GM(1,1) model and the GM(1,1) model with self-memory principle (SMGM(1,1) model) were used to predict the incidence rates of the two notifiable infectious diseases based on statistical incidence data. Both simulation accuracy and prediction accuracy were assessed to compare the predictive performances of the three models. The best-fit model was applied to predict future incidence rates.

**Results:**

Simulation results show that the SMGM(1,1) model can take full advantage of the systematic multi-time historical data and possesses superior predictive performance compared with the linear model and the conventional GM(1,1) model. By applying the novel SMGM(1,1) model, we obtained the possible incidence rates of the two representative notifiable infectious diseases in China.

**Conclusion:**

The disadvantages of the conventional grey prediction model, such as sensitivity to initial value, can be overcome by the self-memory principle. The novel grey self-memory coupling model can predict the incidence rates of infectious diseases more accurately than the conventional model, and may provide useful references for making decisions involving infectious disease prevention and control.

## Introduction

Prevention and control of infectious diseases is an increasingly important public health issue. After World War II, developments in preventive medicine, clinical medicine, and preclinical medicine have served major roles in preventing and controlling infectious diseases. Many acute or chronic infectious diseases have been effectively controlled by the use of antibiotics and vaccines [1], [2]. However, the chronicity, arduousness, and complexity of infectious disease prevention and control are often ignored, which has resulted in the emergence of new pathogens with changes in the environment and the expansion of domestic and international communication [3], [4]. The World Health Organization has declared that epidemic factors spreading infectious diseases include global population movement, emergence of drug-fast pathogenic microorganisms, global climatic variation, social unstable factors, and flaws in health management [5]. Strategies to prevent and control infectious diseases are particularly urgent in developing countries with a weak economy, poor sanitary condition, large population density, and significant international and domestic population movement [6], [7]. Previous experiences have shown that accurate forecasts and analyses of the future trend of infectious diseases can realize timely epidemic detection and prevention. Establishing long-term prevention strategies can lay the foundation for early warning of diseases and provide a theoretical basis for drafting prevention strategies and solutions. Therefore, early warning and forecast of infectious diseases is important for health care and public hygiene management.

A statistical analytical method that combines mathematics and infectious disease epidemiology is used to predict the emergence of infectious diseases. This method has been widely applied for early warning and forecast of all types of infectious diseases. Model-based prediction methods for epidemics have been a major research focus in public health. Studies commonly combine statistical analysis (i.e., regression analysis [8] and time series analysis [9]) and mathematical modeling (i.e., epidemic dynamic model [10], [11]) to forecast epidemic regularity and development trends based on data related to infectious diseases. These methods have been actively researched for their utility in generating early warnings against disease outbreaks [12], [13]. In particular, prediction of disease incidence rates is a popular topic of research. Disease forecasts can provide important references for public hygiene management, and serve as effective resources for planning, prevention and control. With the development of powerful prediction theories, statistical regression models [14]–[16], time-sequences models [17], [18], discrete time stochastic model [19], age-structured epidemic model [20], grey system models [21], [22], Markov chain models [23], [24], and artificial neural network models [25], [26] have been applied to predict future epidemiological trends. However, common statistical prediction methods have limitations. For example, large samples of historical data that follow a certain representative statistical distribution are required. The evolutionary path of an infectious disease is influenced by uncertainties and characterized as a grey system. Hence, the occurrence and prevalence of an infectious disease can be regarded as a typical dynamic variable grey system. Importantly, grey system models are practical because data sample size and probability distribution are not strictly required.

When dealing with small samples and incomplete information, traditional methods (i.e., probability statistics and fuzzy mathematics) show limitations [27], [28] such as requirement of a large amount of statistical data and a structuralized system. To overcome these disadvantages, Deng first proposed the grey systems theory in 1982 to study the uncertainty of systems with unclear statistical distribution [29]. The grey model uses the accumulated generating operation to process raw data to reduce their degree of stochasticity and to increases their regularity[30], [31]. As the simplest model, the GM(1,1) model (univariate first-order differential equation) is especially appropriate for predicting the overall development tendency of the dynamical system (i.e., approximate exponential increasing tendency and exponential decreasing tendency). It has been broadly applied in numerous fields, such as economy and management, industry and agriculture, medicine and health, and engineering sciences [32]–[37]. However, the conventional GM(1,1) model essentially belongs to the initial value solving problem of differential equations which only meet the initial condition at one point, i.e., the observed values at one moment. Accordingly, the original dynamical differential equation has the limitation of being sensitive to initial values, and that becomes a disadvantage when historical information is not fully available. Recently, many scholars have focused on improving the prediction performance of the grey prediction models. The self-memory principle is one of the most important methods to enhance the precision of a model.

On the basis of retrieved modeling methods, the self-memory principle of dynamic system was first proposed by Cao in 1993 [38]. As a statistically dynamic method to solve problems of nonlinear dynamic systems, it successfully integrated determinism and random theories with mathematics [39]. The self-memory principle can retrieve ideal nonlinear dynamic models from practical observational data. It overcomes the weakness of being sensitive to initial values for differential equations, as well as the limitation of irrelevance to the mechanism modeling due to utilization of historical materials. The method is a breakthrough for numerical solution of traditional initial-value problems and statistical approaches. This novel prediction model combines the advantages of the self-memory principle and the grey GM(1,1) model by coupling their prediction methods. Its excellent predictive performance lies in the fact that the weakness of conventional GM(1,1) model, i.e., sensitivity to initial value, can be overcome by using a multi-time-point initial field instead of a single-time-point initial field. The concept has been utilized increasingly in time series forecasting in multiple fields, such as meteorology, engineering, and economics [40], [41]. In recent years, some scholars have attempted to introduce the self-memory principle into certain basic grey prediction models [42]–[44]. However, these published research methods have been applied mainly in the fields of meteorology, hydrography, and engineering science, and only minimally in medicine and public health.

In the fields of epidemiology and public hygiene, the occurrence and prevalence of an infectious disease are commonly accompanied by irregular individual fluctuations due to many internal and external unstable factors. Hence, the spread and prevalence of an infectious disease can be regarded as a dynamical variable grey system with stochastic fluctuation. The grey prediction models have been effectively utilized for forecast and analysis of the morbidity and mortality of epidemics, such as parasitosis and phymatosis [21], [37]. In this paper, in light of the uncertainty features of infectious diseases, two representative infectious diseases (dysentery and gonorrhea) among categories A and B of notifiable diseases in China were selected to predict their incidence rates by coupling the GM(1,1) model and the self-memory principle. Although most infectious diseases have been successfully controlled in China, handling of epidemics of some infectious diseases in certain regions are not going well [22]. Dysentery is a major public health issue in many countries in the world [45]. Transmission of dysentery is fecal-oral, which may involve polluted food, water, daily contact, and flies. Despite the fact that the incidence of intestinal infectious diseases has declined considerably in recent years worldwide, the incidence of dysentery remains high in developing countries. Dysentery is one of the most common epidemics in overcrowded areas with inadequate sanitation. Notably, dysentery is a recurrent challenge in many parts of the world. In addition, gonorrhea, a bacterial infection caused by *Neisseria gonorrhoeae*, is a highly communicable sexually transmitted infection and, due to its short incubation period, may serve as an indicator of recent risky sexual behavior in symptomatic cases [46]. Furthermore, in developing countries, sexually transmitted diseases and their complications are among the top five conditions for which adults seek medical care. These conditions may cause acute or chronic symptoms as well as delayed sequels such as infertility, ectopic pregnancy, cervical cancer and premature fatalities among infants and adults. Consequently, dysentery and gonorrhea rank top in categories A and B of notifiable diseases in China.

In this study, prediction performances of the SMGM(1,1) model, the conventional GM(1,1) model and the linear model were compared. The model with the best fit was then utilized to deduce emerging epidemic tendencies. We propose that the grey self-memory coupling prediction model is appropriate for forecasting the incidence rates of infectious diseases in China. Results of our analyses may provide effective guidance in the decision-making process for the prevention and control of epidemics.

## Materials and Methods

### Data sources

In this study, the incidence rates of two types of notifiable infectious diseases (dysentery and gonorrhea) were investigated. The incidence rates of the two infectious diseases were obtained from public governmental statistical data, published by the China Health Statistical Yearbook of 2013 [47]. Since the Chinese Centre for Disease Control and Prevention was founded in 2002, and it experienced the ordeal of the SARS epidemic the following year, only statistical data from 2004 to 2012 were selected for modeling analysis.

### Methods

The linear model was formulated as y = mx+b. Detailed principles of modeling analysis and accuracy assessment of the grey self-memory coupling prediction method have been described previously by Wu et al. [31] and Cao et al. [48]. Excel software, Grey system software 3.0 and MATLAB software 7.0 were utilized for modeling and simulation.

#### A. The principle of grey self-memory coupling prediction

After the accumulated original statistical data are generated and the moving average is calculated, the grey system GM(1,1) model can weaken the randomness of the original data and generate regular cumulative data. Consequently, the prediction model can be approximated by the solution of a linear first-order differential equation. Nevertheless, the self-memory principle not only emphasizes the overall exponential development tendency of the dynamical system, but also its individual stochastic fluctuations. After introducing the memory function, which contains historical information, into the system's dynamic differential equation, the equation can be transformed into a difference-integral equation, called self-memorization equation, by defining the inner product in Hilbert space. Because the systematic self-memorization equation contains multiple time-point initial fields instead of only single time-point initial field, the weakness of being sensitive to the initial value of the original dynamic differential equation can be overcome. By studying systematic inner memorability, the systematic overall exponential development tendency with individual stochastic fluctuations can be modeled and predicted. The superiority of the self-memory principle lies in the fact that the systematic predictive ability can be improved by not only combining dynamics calculations and estimating parameters of historical data, but also extracting systematic information from historical data in statistics.

#### B. Model construction

For our modeling analysis, the incidence data of the two infectious diseases from 2004 to 2011 were taken as the modeling samples (i.e., original series 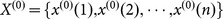). Furthermore, the incidence data of the year 2012 were selected as the testing samples for the prediction test.

First, the general procedure for a conventional GM(1,1) model was derived as follows:

Step 1. Assume that the sequence 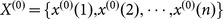 is an original non-negative data sequence, where 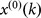 is the time series data at time 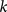. The sequence 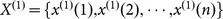 is the first-order accumulated generation sequence of 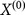, where 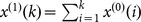, 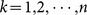.

Step 2. The basic form of the GM(1,1) model is defined as 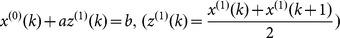
where the parameters 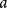 and 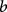 are called developing and grey input coefficients, respectively. Let sampling time 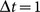; then, by the least square method, the parameters 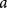 and 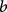 can be obtained as



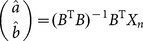
where



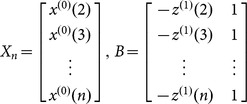



Step 3. The whitenization equation of the GM(1,1) model is given by 
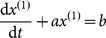
. By making the initial value 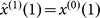, the time response sequence of the GM(1,1) model is given by
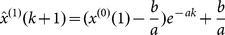
(1)and the simulative value of sequence 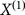 can be obtained from **Eq. 1** accordingly. Consider the inverse accumulated generation



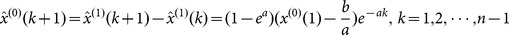



Thus, the simulative value of sequence 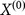 can be obtained.

The procedure followed to develop a novel SMGM (1,1) model is as follows:

Step 1. Determining the self-memory dynamic equation.

Let 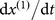 in the whitenization differential equation of the GM(1,1) model be 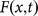. Then,
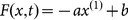
(2)


The differential equation 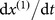, determined by **Eq. 2**, is considered systematic self-memory dynamic equation of the grey self-memory coupling model:
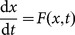
(3)where 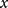 is a variable, 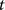 is time interval series, and 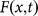 is the dynamic kernel. Then, a memory function 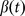 was introduced and an inner product in the Hilbert space was defined:



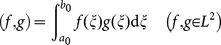
(4)Step 2. Deducing the difference-integral equation.

Let one time set 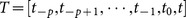, where 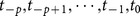 is historical observation time, 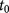 is predicted initial time, 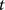 is coming prediction time, the retrospective order of the equation is 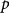 and time sampling interval is 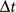. After applying the above inner product operation into **Eq. 3** and supposing that variables 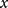,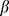 are continuous, differentiable, and integrable, the analytic formula of **Eq. 3** is obtained as 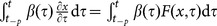, that is,
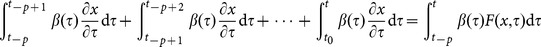
(5)


For every integral term in the left-hand side of **Eq. 5**, after applying calculus and performing integration by parts, applying the median theorem, and performing algebraic operations, a difference-integral equation is deduced as:
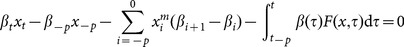
(6)where 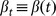, 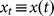, 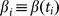, 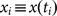, 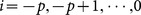, and mid-value 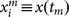, 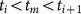.

Step 3. Discretizing the self-memory prediction equation.

Let 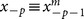 and 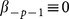, **Eq. 6** can be converted into
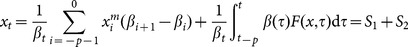
(7)which is called self-memory equation with the retrospective order 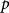. The first term 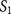, defined as the self-memory term in **Eq. 7,** denotes the relative contributions of historical data at 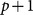 times to the value of variable 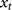. The second term 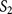, defined as the exogenous effect term, is the total contribution of the function 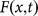 in the retrospective time interval 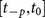. **Eq. 7** emphasizes serial correlation of the system by itself, i.e., the self-memory characteristic of the system. Therefore, **Eq. 7** is the self-memory prediction equation of the system. If integral operation is substituted by summation and differential is transformed into difference in **Eq. 7**, then the mid-value 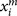 is replaced by two values of different times, namely, 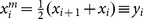. By taking equidistance time interval 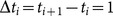, and merging 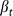 and 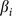 together, the self-memory equation of discrete form is shown as follows:
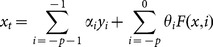
(8)where 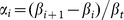,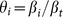. 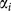 and 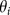 are called memory coefficients, and 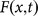 is determined by the dynamic kernel 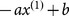 of the GM(1,1) model.

Step 4. Solving the self-memory prediction model.

Assuming that there are 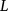 items of historical data, the memory coefficients 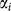 and 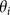 can be estimated by the least square method. Let
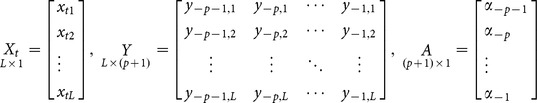


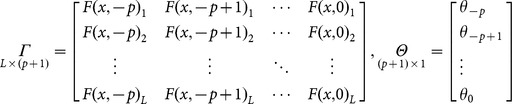



Then, **Eq. 8** can be expressed in matrix form as follows:
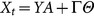
(9)


Let 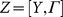, 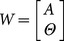, then **Eq. 9** turns into 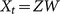, thereby 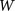 is obtained by the least square method: 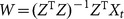. When the memory coefficients matrix 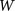 is obtained, the simulation and prediction of original data sequence 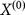 can be performed. For the simulated and predicted value 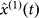 of the accumulated generation sequence in grey self-memory coupling model, its inverse accumulated value 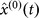 can be obtained as follows:
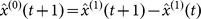
where 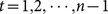 and 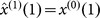.

#### C. Modeling simulation and prediction accuracy assessment

Simulation and prediction accuracy is an important criterion for evaluating prediction models. Accuracy test must be performed to evaluate the rationality and reliability of prediction models before extrapolation and application. Consequently, two popular test criteria such as variance ratio and small error probability [22] were used to compare the accuracy of different prediction models, as shown in Table 1.

**Table 1:** List of variance ratio and small error probability obtained in the accuracy test.

Modeling accuracy class	Test index	
	Variance ratio C	Small error probability p
1st level (superior)	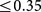	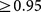
2nd level (qualified)	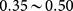	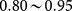
3rd level (marginal)	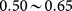	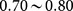
4th level (disqualified)	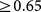	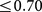


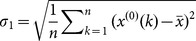
 and 
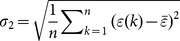
 are the mean square error of original values and residual error, respectively. Given 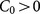, if the variance ratio 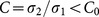, then the model is considered to pass through the variance ratio test [22].

In the same way, given 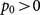, if the small error probability 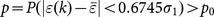, then the model is supposed to pass through the small error probability test [22].

The absolute percentage error at time 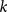 is denoted by 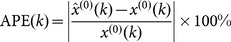, and the mean absolute percentage error at all times is defined as 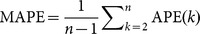. Accordingly, comparisons between the actual values and simulative values derived from each prediction models can be analyzed using 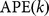 and 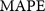.

#### D. Programming procedure of MATLAB software

**Figure 1:**
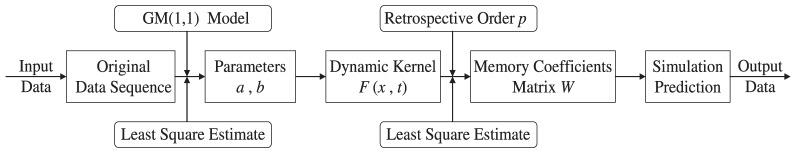
Programming procedure for the SMGM(1,1) model.

The calculation was performed, as mentioned above, with the help of MATLAB software for computational efficiency. The programming procedure for the SMGM(1,1) model is shown in Fig. 1.

### Data analysis

The incidence rates of two representative infectious diseases (dysentery and gonorrhea) from 2004 to 2012 in China were adopted to demonstrate the practicability and effectiveness of the proposed SMGM(1,1) model and its superiority to the linear model and the conventional GM(1,1) model. On the whole, the time series of incidence rates of dysentery and gonorrhea all showed an obvious exponential decreasing tendency. They were accompanied by some irregular individual fluctuations due to unstable changes imposed by social and economic factors. Therefore, two SMGM(1,1) models were established to model and predict the incidence rates, and were compared with their corresponding linear models and conventional GM(1,1) models. When performing the modeling analysis, the incidence rates of the first eight time-points were taken as modeling samples, and the data of the ninth time-point was selected as the test sample for the prediction test. In accordance with the principles and the steps mentioned above, two SMGM(1,1) models were established to forecast incidence rates. The value of retrospective order was uniformly determined as 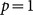 by trial calculation under the principle of minimum error. 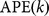 and MAPE were used to compare the actual values with the simulated values to evaluate the predictive performance compared to linear models and conventional GM(1,1) models.

## Results

### Forecasting the incidence rates of dysentery

Based on the incidence data of dysentery from 2004 to 2011, the differential equation of conventional GM(1,1) model can be formulated as follows:
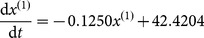
(10)


Let the right-side term of **Eq. 10** be the dynamic kernel 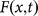. Then, the systematic self-memory dynamic equation 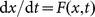 of SMGM(1,1) model is obtained. After applying the inner product operation **(4)** into 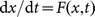, then the analytic formula is obtained as 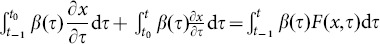. According to the modeling steps mentioned above, a difference-integral equation is deduced as 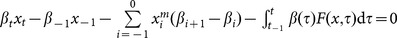, and the self-memory prediction equation is obtained as 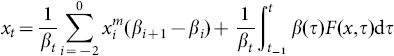. After the integral operation is substituted by summation and the differential is transformed into difference, the discrete form of self-memory equation for dysentery incidence can be expressed as 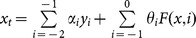, where 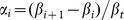, 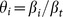. Using the least square estimation method, the memory coefficients matrix can be obtained as
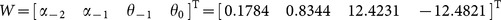



The simulated values and errors of the linear model, the conventional GM(1,1) model and the SMGM(1,1) model are presented in Table 2. The variance ratio and small error probability of the three models are all at the first level, as shown in Table 1. Since the three models passed the simulation accuracy assessment, they could be used to perform predictions. From the 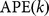 and 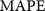 of fitting values, as shown in Table 2, the simulated precision of the novel SMGM(1,1) model is markedly superior to that of the other two models. Considering the incidence rate in 2012, the SMGM(1,1) model also exhibits better single-step predictive performance compared with the other models. Using the SMGM(1,1) model, the next incidence rate of dysentery in China is predicted to be 14.10 per 100,000.

**Table 2:** Simulated values and errors of the linear model, the GM(1,1) model and the SMGM(1,1) model for the incidence rate of dysentery (1/10^5^).

		Linear model		GM(1,1) model		SMGM(1,1) model	
Year	Original Value	Simulative value	APE	Simulative value	APE	Simulative value	APE
2004	38.30	37.91	1.01%	38.3	—	—	—
2005	34.92	34.77	0.44%	35.38	1.32%	—	—
2006	32.36	31.62	2.28%	31.22	3.52%	32.40	0.12%
2007	27.99	28.47	1.73%	27.55	1.57%	27.45	1.93%
2008	23.43	25.33	8.10%	24.32	3.80%	24.60	4.99%
2009	20.45	22.18	8.47%	21.46	4.94%	19.93	2.54%
2010	18.90	19.03	0.71%	18.94	0.21%	18.42	2.54%
2011	17.74	15.89	10.44%	16.71	5.81%	18.06	1.80%
MAPE			4.15%		3.02%		2.32%
2012	15.40	12.74	17.26%	14.75	4.22%	15.12	1.82%

There is no simulated value for the first two time-points because of the retrospective order 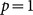.

### Forecasting the incidence rates of gonorrhea

Based on the incidence data of gonorrhea from 2004 to 2011, the differential equation of the conventional GM(1,1) model is formulated as follows:
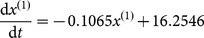



Then, the prediction equation of gonorrhea incidence can be similarly obtained by using the same formula above, where the memory coefficients matrix is
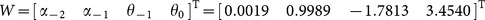



The simulated values and errors of the linear model, the conventional GM(1,1) model and the SMGM(1,1) model are presented in Table 3. All models passed the simulation accuracy assessment, and the simulated and single-step predictive precisions of the SMGM(1,1) model were also superior to other two models. Thus, the next incidence rate of gonorrhea in China is estimated at 6.05 per 100,000 based on the SMGM(1,1) model.

**Table 3:** Simulated values and errors of the linear model, the GM(1,1) model and the SMGM(1,1) model for the incidence rate of gonorrhea (1/10^5^).

		Linear model		GM(1,1) model		SMGM(1,1) model	
Year	Original Value	Simulative value	APE	Simulative value	APE	Simulative value	APE
2004	17.34	15.30	11.78%	17.34	—	—	—
2005	13.79	14.10	2.22%	13.67	0.87%	—	—
2006	12.14	12.90	6.23%	12.29	1.24%	12.14	0.00%
2007	11.08	11.70	5.55%	11.05	0.27%	11.11	0.27%
2008	9.90	10.49	6.00%	9.93	0.30%	9.85	0.51%
2009	9.02	9.29	3.03%	8.93	1.00%	8.96	0.67%
2010	7.91	8.09	2.31%	8.03	1.52%	8.03	1.52%
2011	7.31	6.89	5.71%	7.21	1.37%	7.28	0.41%
MAPE			5.36%		0.94%		0.56%
2012	6.82	5.69	16.55%	6.49	4.84%	6.64	2.64%

There is no simulated value for the first two time-points because of the retrospective order 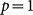.

**Figure 2:**
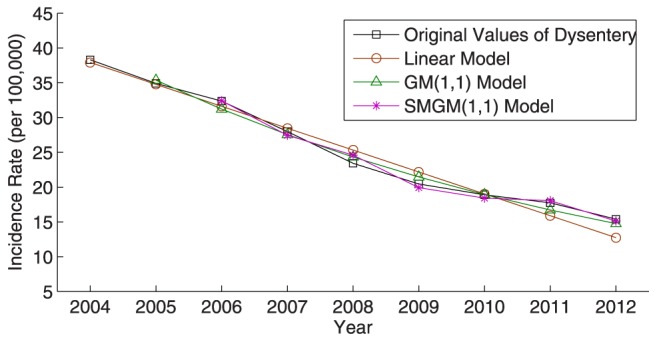
Comparison of incidence rates and simulated values among the three different prediction models for dysentery.

**Figure 3:**
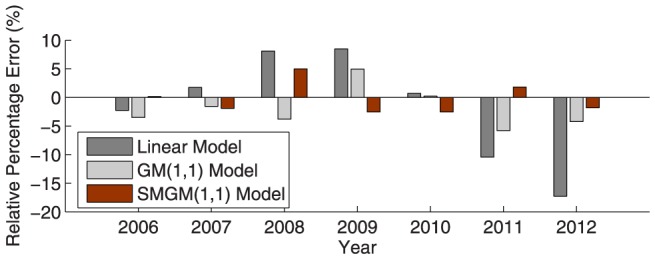
Relative percentage error distribution of the three different prediction models for dysentery from 2006 to 2012.

**Figure 4:**
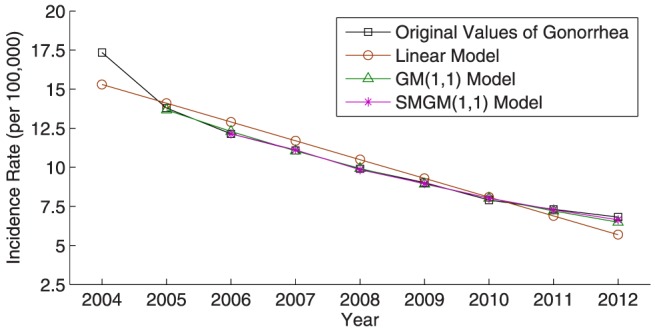
Comparison of incidence rates and simulated values among the three different prediction models for gonorrhea.

**Figure 5:**
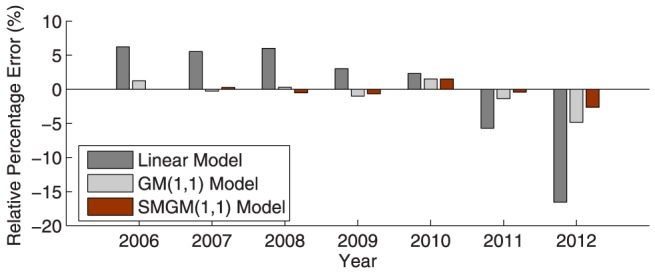
Relative percentage error distribution of the three different prediction models for gonorrhea from 2006 to 2012.

Furthermore, Figs. 2–5 illustrate the fitting results of the simulated curves obtained by the three compared models with the original incidence curves of dysentery and gonorrhea, and their corresponding comparison results of relative percentage error distribution. From the comparative analysis, the prediction accuracy of the proposed SMGM(1,1) model is remarkably higher than that of the linear model and the conventional GM(1,1) model. Consequently, the proposed SMGM(1,1) model can better catch the tendency of integral development and individual variation of original data, and is a reliable and stable prediction model for predicting the future development tendency of infectious diseases.

## Discussion

The GM(1,1) model, as a basic and typical grey system prediction model, mainly reflects the statistical laws of diseases by processing the corresponding statistical data. Through conversion of the original sequence, the model establishes a regression equation based on the regular generated sequence. Consequently, prediction of the dynamic development trend of diseases can be conducted using the regression equation. The GM(1,1) model is gradually developing into a common analytical method for both medicine and public health [22], [36], [37]. The grey self-memory coupling model is established on the basis of the conventional GM(1,1) model by combining it with the self-memory principle of dynamic systems. The coupling model can reflect the macroscopic development laws of disease systems based on the GM(1,1) model, and further explores their microscopic fluctuating laws with the help of the self-memory principle. The excellent prediction performance of the coupling model is supported by the systematic self-memorization equation containing multiple time-point initial fields instead of only single time-point initial fields. The equation overcomes the weakness of being sensitive to initial values of the conventional GM(1,1) model and takes full advantage of the system information contained in historical data [42].

In this paper, the conventional GM(1,1) model and the SMGM(1,1) model were utilized to predict the incidence rates of three representative infectious diseases in China. Analysis of simulation results indicated that the SMGM(1,1) model possesses more superior predictive performance than the conventional GM(1,1) model. Therefore, the SMGM(1,1) model was selected to predict the future incidence rates of three infectious diseases in China. The incidence rate is the most straightforward and objective index, which evaluates the effect of prevention and control measures, because the numerator of the incidence rate is the new morbidity number within a time of resignation. The model significantly reduces prediction error of future incidence rates of infectious diseases. The combination of self-memory component and the grey system model can obtain encouraging prediction performances. This coupling model is a practical forecasting tool for infectious diseases with characteristics of stable epidemic factors, because its requirements of the original data are not as strict as those of other common statistical models. Therefore, the novel coupling prediction model can be used to predict the regularity of occurrence and development of infectious diseases and to identify their dynamic tendencies, thus providing a scientific basis for the prevention and control of infectious diseases.

Results of our simulations show that the novel grey self-memory coupling prediction model has improved the prediction accuracy of incidence rates, especially for short-term prediction. However, the model has certain limitations. First, when the grey self-memory coupling model is applied to long-term prediction, the prediction accuracy may decline. For this reason, the model needs to be further amended [49]. Over time, unknown disturbance factors may enter the system and exert unpredictable influences. Therefore, to forecast the long-term incidence rates of infectious diseases, the latest data should be utilized to amend the model to generate a new information model. Old data, which have minor effects on system development, may be discarded to improve the prediction accuracy. The spread of infectious diseases is inevitably influenced by natural, social, and environmental factors [50]. As a result, the most suitable model should be constructed by synthetically considering and systematically analyzing relevant influencing factors [22]. Prevention and early warning of infectious diseases can then be performed successfully using the appropriate prediction models.
